# Whole-Genome Resequencing Analysis of Copy Number Variations Associated with Athletic Performance in Grassland-Thoroughbred

**DOI:** 10.3390/ani15101458

**Published:** 2025-05-18

**Authors:** Wenqi Ding, Wendian Gong, Tugeqin Bou, Lin Shi, Yanan Lin, Xiaoyuan Shi, Zheng Li, Huize Wu, Manglai Dugarjaviin, Dongyi Bai

**Affiliations:** 1 Key Laboratory of Equus Germplasm Innovation (Co-Construction by Ministry and Province), Ministry of Agriculture and Rural Affairs, Hohhot 010018, China; dingwenqi0331@gmail.com (W.D.); gongwendian1996@outlook.com (W.G.); tvgqin@gmail.com (T.B.); 19832607527@163.com (L.S.); linyanan@emails.imau.edu.cn (Y.L.); xiaoyuans2021@163.com (X.S.); lzheng0511@sina.com (Z.L.); whz020419@163.com (H.W.); dmanglai@163.com (M.D.); 2 Inner Mongolia Key Laboratory of Equine Science Research and Technology Innovation, Inner Mongolia Agricultural University, Hohhot 010018, China; 3 Equus Research Center, College of Animal Science, Inner Mongolia Agricultural University, Hohhot 010018, China

**Keywords:** CNV, Grassland-Thoroughbred, Xilingol horses, whole-genome, athletic selection traits

## Abstract

Copy number variation (CNV) is an important source of genetic variation. However, studies utilizing whole-genome sequencing to investigate CNVs in horse populations and their effects on traits remain relatively limited. This study aims to address the lack of research on the impact of copy number variation (CNV) on racing performance in horse populations, providing new insights for locally bred racing breeds. We analyzed 60 offspring derived from the crossbreeding of Thoroughbred horses and Xilingol horses. These horses were temporarily named “Grassland-Thoroughbred” and were divided into two groups: 30 racing horses and 30 non-racing horses. A total of 89,527 CNVs were identified. After merging overlapping CNVs, 982 copy number variation regions (CNVRs) were recognized, among which the racing horse group (RH) had 29 unique CNVRs, while the non-racing horse group (NR) had 4 unique CNVRs. In addition, a total of 195 genes overlapping with CNVRs were identified. Transcriptomic analysis revealed 120 differentially expressed genes, with *MTPN* expressed in both CNVR-overlapping genes and mRNA. Both CNVR-overlapping genes and differentially expressed genes were enriched in the MAPK signaling pathway; CNV may affect gene expression through gene dosage effects or regulatory mechanisms. Using Vst statistical analysis, we further screened candidate CNVRs in autosomes that exceeded the 95% differentiation threshold between the RH and NR populations. Several key genes associated with energy metabolism and muscle function were identified, including *AGT*, *IGFN1*, *IMMPL2*, *SLC41A3*, *AOX4*, and *ACAD11*. These findings provide new insights into the genetic structural variation in racing performance and adaptability, fill the gap in CNV studies in the genomics of Grassland-Thoroughbred horses, and offer valuable genomic data for optimizing breeding strategies in native racing horse populations.

## Simple Summary

This study investigates the impact of copy number variation (CNV) on racing performance in horse populations, focusing on 60 “Grassland-Thoroughbreds”. A total of 89,527 CNVs were identified, resulting in 982 CNVRs. The racing horse group (RH) had 29 unique CNVRs, while the non-racing horse group (NR) had 4. Transcriptomic analysis revealed 120 differentially expressed genes, with *MTPN* overlapping with CNVRs. Both CNVRs and differentially expressed genes were enriched in the MAPK signaling pathway. Vst analysis identified key genes related to energy metabolism and muscle function, such as *AGT*, *IGFN1*, *IMMPL2*, *SLC41A3*, *AOX4*, and *ACAD11*. These findings offer new insights into genetic variation in racing performance and provide valuable data for improving breeding strategies.

## 1. Introduction

Horse racing is a major competitive sport and industry worldwide, widely conducted in over 70 countries and regions, involving more than 500,000 racehorses. The total prize money for global racing events exceeds 3.3 billion euros, while annual horse racing betting revenue surpasses EUR 115 billion [1,2]. Internationally renowned events, such as the Melbourne Cup, Kentucky Derby, and Japan Cup, not only enjoy a high reputation but also have a profound impact on the local economy, employment growth, and the development of the entertainment industry [1,2,3]. The horse racing industry encompasses breeding, training, event operations, and related services, with significant economic and social value. Therefore, breeding improvement and genetic research on racehorse breeds are crucial [4].

The Mongolian horse is one of the oldest horse breeds in the world, renowned for its exceptional endurance, ability to adapt to extensive farming conditions, and strong disease resistance [5]. As an important local horse breed resource in China, the Mongolian horse is often used as the foundation mare for improving other horse breeds. The Xilingol horse is a new breed developed through systematic crossbreeding based on the Mongolian horse, and it has gained attention for its faster speed and improved conformation [6]. To further enhance the athletic performance of local horse breeds, Thoroughbred horses were introduced and crossbred with Xilingol horses starting in 1995. After nearly 30 years of selective breeding, the “Grassland-Thoroughbred” was developed, featuring excellent speed and conformation. Due to the sunflower symbol on its hindquarters, it is also known as the “Sunflower Horse”. This breed of horse is primarily engaged in middle-to long-distance races. As horse genetic improvement progresses, the focus of research has gradually shifted from solely addressing conformation and speed to genomic studies.

Copy number variation (CNV) is one of the important sources of genetic diversity and an essential contributor to phenotypic variation [7,8]. CNVs are structural variations involving DNA fragments of 1 kb or larger, typically resulting from deletions, insertions, or duplications, and they vary among individuals and species [9,10,11]. Widely distributed across the genome, CNVs can influence gene function through mechanisms such as altering gene dosage or transcript structure, thereby regulating phenotypic plasticity in organisms [12,13]. The total number of nucleotides affected by CNVs has surpassed that of single-nucleotide polymorphisms [14]. Since this discovery, CNVs have been widely recognized as a genomic variation that may have a significant impact on phenotypes. Studies have shown that heritable CNVs can influence both Mendelian traits and complex disease traits [15].

This study aims to explore the genetic basis of specific uses of the Grassland-Thoroughbred horse, focusing on the genetic differences between racehorses and non-racehorses and revealing genomic characteristics associated with athletic performance. Through in-depth analysis of genomic data and transcriptomic data of 30 racehorses and 30 non-racehorses, we investigate the genetic mechanisms underlying athletic traits in the Grassland-Thoroughbred horse. In line with the breeding goals of the Grassland-Thoroughbred, potential target genes related to athletic performance were identified. These findings provide a theoretical foundation for the breeding and genetic improvement of the Grassland-Thoroughbred horse. Our preliminary results not only expand the CNVR dataset in equine genomics but also offer valuable genomic insights for optimizing breeding strategies in local racing horse populations. Additionally, it contributes to enhancing the genetic diversity of Chinese horse breeds and supports the conservation and sustainable utilization of equine resources.

## 2. Materials and Methods

### 2.1. Sample Collection

The Grassland-Thoroughbred horses used in this study are descendants of multiple generations of crossbreeding between the Xilingol horse and Thoroughbred. These individuals were obtained from Inner Mongolia Grassland Thoroughbred Horse Breeding Co., Ltd. (Xilinhot, China), which holds comprehensive pedigree records and breeding line information for hybrid horses. According to the pedigree records, individuals with higher degrees of relatedness have been excluded. The experimental groups included a non-racing Grassland-Thoroughbred horse group (NR group, *n* = 30), which had no competitive training or racing experience, and a racing Grassland-Thoroughbred horse group (RH group, *n* = 30), which demonstrated competitive performance. The performance of horses in the RH group was evaluated through multiple regional competitions, and individuals who consistently achieved top rankings were classified as high-performance horses (Supplementary Table S1). Blood samples were collected from the jugular vein of each horse by professional technicians and placed in EDTA anticoagulant tubes. The samples were then aliquoted, rapidly frozen in liquid nitrogen, and stored at −80 °C to ensure sample integrity for subsequent experiments.

### 2.2. Library Construction and Sequencing

The DNA extracted from the blood samples of each horse was fragmented using ultrasound. Following the manufacturer’s protocol, sequencing libraries were constructed using the NEBNext^®^ Ultra™ DNA Library Prep Kit for Illumina (NEB, Ipswich, MA, USA), with unique index codes added to each sample [16]. The purified libraries were quality-assessed using the Agilent 5400 system (Agilent, Santa Clara, CA, USA) to evaluate the size distribution and purity of the DNA fragments, ensuring they met the requirements for sequencing. Qualified libraries were then sequenced on the Illumina HiSeq 4000 platform (Novogene Co., Ltd., Beijing, China) with paired-end sequencing (PE150) to obtain high-quality sequencing data.

### 2.3. Data Filtering and Sequence Alignment

Raw data were processed using fastp software (version 0.20.0) to remove reads with unrecognized nucleotides (N) ≥ 5%, Phred quality scores lower than 15, base quality < 20%, presence of adapter sequences (allowing mismatches ≤ 3%), and a length shorter than 15 bases, thereby obtaining clean reads [17,18]. The clean reads were then aligned to the horse reference genome (https://www.ncbi.nlm.nih.gov/datasets/genome/GCF_002863925.1/, accessed on 27 November 2020) using BWA software (version 0.7.15) [19]. The alignment results in SAM format were converted to BAM files using samtools (version 1.19.2) [20], followed by sorting. Potential PCR duplicates were removed using the MarkDuplicates function of Picard software (version 1.119) [21].

### 2.4. Phylogenetic Analysis

The PIHAT (Proportion of Identical by Descent) values of SNPs from the PLINK software (version 1.9) were used to estimate the homozygosity proportion between horses. Based on these values, a genetic distance matrix was generated for pairwise individuals [22].

### 2.5. CNV and CNVR Detection

CNV detection was performed using CNVnator (version 0.3.3) [23], with a sliding window size set to 100 bp to identify CNVs in the horse genome samples. After detection, raw CNVs for each horse were subjected to quality control. The filtering criteria were as follows: CNVs with a *p*-value < 0.05, size > 1 kb, and a q0 (the fraction of zero-quality mapping reads) < 0.5 were retained for downstream analysis. Copy number variation regions (CNVRs) were determined by aggregating overlapping CNVs identified in different individuals. To minimize false positives, only CNVRs observed in seven or more individuals within each breed were considered for further analysis [24]. After obtaining the common CNVRs for each group, further analysis was conducted to identify differential CNVRs between the NR and RH groups.

### 2.6. Genomic Selection Signals Based on CNVRs

Highly differentiated CNV regions between the RH and NR groups were evaluated using the Vst statistic and analyzed in combination with the *t*-test. The Vst value ranges from 0 to 1, with a higher value indicating greater differentiation in copy number variation between the two groups for a given region, while lower values suggest smaller differences. CNVRs with a Vst value exceeding the 95% threshold were considered candidate CNVRs. These candidate regions were then subjected to functional enrichment analysis to identify potentially important biological processes or pathways associated with the observed selection signals.

### 2.7. RNA-Seq Library Construction

Three blood samples from both the competition and non-competition groups were randomly selected to extract total RNA. After assessing the RNA purity and integrity using the Agilent Bioanalyzer 2100 system (Agilent Technologies, Santa Clara, CA, USA), the chain-specific library preparation method was employed. RNA sequencing (RNA-seq) libraries were constructed using the NEBNext^®^ Ultra™ Directional RNA Library Preparation Kit. The libraries were subsequently purified using the AMPure XP system (Beckman Coulter, Beverly, MA, USA). RNA-seq libraries were sequenced on the Illumina HiSeq 2500 platform, generating 150 bp paired-end sequencing reads.

### 2.8. Quality Control and Alignment of RNA Sequencing Data

The raw data after sequencing were subjected to quality control using Fastp. The quality control criteria included removing adapter sequences, excluding reads with ≥10% unrecognized nucleotides (N), and filtering out low-quality reads with a quality score ≤ Q20. Subsequently, the clean data were aligned to the horse reference genome using Hisat2 (version 2.0.4) [25]. The alignment results were converted to the desired format using Samtools, and transcript reconstruction was performed using StringTie (version 2.0) [26] to generate transcript annotation information. Next, gene expression information was extracted using Ballgown (version 2.16.0) [27] in R, and differential gene expression analysis was performed using DESeq2 (version 1.44.0) [28]. The criteria for screening differentially expressed genes (DEGs) were set as |log2(Fold change)| > 1 and FDR ≤ 0.05, ensuring the statistical reliability of the results.

### 2.9. Functional Enrichment Analysis and QTL Association Analysis

The overlapping genes of CNVRs and differentially expressed genes were functionally annotated using the DAVID database (https://david.ncifcrf.gov/, accessed on 4 October 2024) to identify candidate genes, including Gene Ontology (GO) and Kyoto Encyclopedia of Genes and Genomes (KEGG) pathway enrichment analysis [29]. FDR < 0.05 was considered statistically significant for identifying enriched biological functions. To investigate whether these candidate CNVRs were associated with athletic traits, the horse QTL database (https://www.animalgenome.org/cgi-bin/QTLdb/EC/index, accessed on 12 November 2024) was accessed and compared with the identified CNVRs. The Bedtools (version 2.26.0) [30] “intersect” command was used to detect overlaps between the identified CNVRs and known QTLs, which may indicate potential links between these genomic regions and performance-related traits in horses.

### 2.10. PCR Validation

To further confirm the accuracy of the inferred CNVs and reduce the false positive rate, 8 CNVRs were randomly selected and validated using qPCR. The *GAPDH* gene was used as a reference to determine the fold change in copy number. qPCR primers were designed using Primer-3, with reference sequences retrieved from GenBank (Supplementary Table S2). The reaction system was set up as follows: 20 μL total volume, which included 10 μL of 2x Maxima SYBR Green/ROX qPCR mix, 2 μL of DNA, 1 μL of forward primer, 1 μL of reverse primer, and 6 μL of water. The reaction conditions were as follows: initial denaturation at 95 °C for 2 min; and denaturation at 95 °C for 40 cycles, followed by annealing at 60 °C for 30 s. The melt curve stage was set to 95 °C for 15 s and 60 °C for 1 min, followed by 95 °C for 15 s to predict the PCR products.

To validate the accuracy of the sequencing results in blood samples from different horse breeds, quantitative real-time PCR (qRT-PCR) was performed for mRNA. The qRT-PCR primers were designed by Shanghai Sangon Biotech (Shanghai, China) (Supplementary Table S2). Total RNA was reverse transcribed into cDNA using the HiScript^®^ II qRT SuperMix for qPCR kit (Vazyme, Nanjing, China), following the manufacturer’s instructions. *GAPDH* was used as the endogenous reference gene [31]. qRT-PCR was conducted using the CFX96 Real-Time PCR Detection System (Bio-Rad, Hercules, CA, USA) with SYBR^®^ Premix Ex Taq™ II (TaKaRa, Tokyo, Japan). Each sample was tested in triplicate to ensure data reliability and accuracy. The experimental results were calculated using the 2^(−ΔΔCt)^ method to assess Ct value changes. Each sample was set up with three biological replicates to ensure data reliability and accuracy.

## 3. Results

### 3.1. CNV Detection

**Figure 1:**
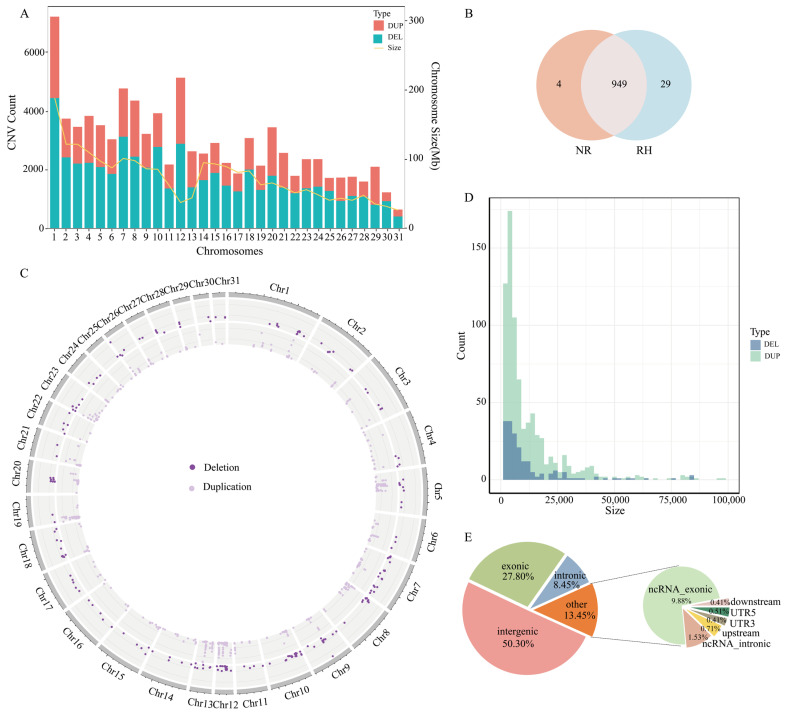
General description of the identified copy number variation regions (CNVRs). (**A**) Distribution of CNVs on autosomes. Light red represents duplication events, cyan blue represents deletion events, and yellow represents the chromosome size of the genome. (**B**) Venn diagram of overlapping CNVRs between the racehorse and non-racehorse populations. NR represents the non-racehorse group, and RH represents the racehorse group. (**C**) Distribution of CNVRs on autosomes. (**D**) Size distribution of CNVRs categorized by status. (**E**) Annotation of the whole-genome CNVs using ANNOVAR.

Using the Illumina paired-end sequencing technology, we obtained high-quality whole-genome resequencing data from 60 Grassland-Thoroughbred horses. To investigate the genetic differences between racehorses and non-racehorses, PIHAT was calculated within the population. The results indicated that the average PIHAT value among the 60 horses was 0.089 (Supplementary Figure S1). The raw data amounted to 3864.02 Gb, while the filtered clean data totaled 3807.14 Gb. After aligning these data to the horse reference genome, the calculated average sequencing depth was 25.63×, with an average mapping rate of 99.19%. This indicates that the sequencing data quality is sufficient for CNV detection (Supplementary Table S3). A total of 89,527 CNVs were detected on the autosomes, including 54,720 deletions and 34,807 duplications (Supplementary Table S4). Among them, Chr 1 had the highest number of detected CNVs, while Chr 31 had the fewest (Figure 1A).

By merging the overlapping CNVs from both groups, a total of 982 CNVRs were identified (Figure 1B) (Supplementary Table S5). These CNVRs were present in at least seven individuals within the same group. The frequency of duplication events was approximately 3.8 times that of deletion events, including 203 deletion regions and 779 duplication regions. The detected CNVRs exhibited considerable length variation, ranging from 1.1 kb to 364.9 kb, with an average length of 14.7 kb. A total of 617 CNVRs (approximately 62.83%) were smaller than 10 kb (Figure 1D). Chr 12 had the highest number of detected CNVRs, with one hundred seven CNVRs, while Chr 30 had the fewest, with only three CNVRs (Figure 1C). NR and RH had four and twenty-nine specific CNVRs, respectively. CNVRs were annotated using ANNOVAR software (https://annovar.openbioinformatics.org/en/latest/user-guide/download/, accessed on 12 June 2023). It was found that 50.3% of the CNVRs were located in intergenic regions, 27.8% in intronic regions, and 8.45% in exonic regions (Figure 1E).

### 3.2. CNVR Functional Enrichment

**Figure 2:**
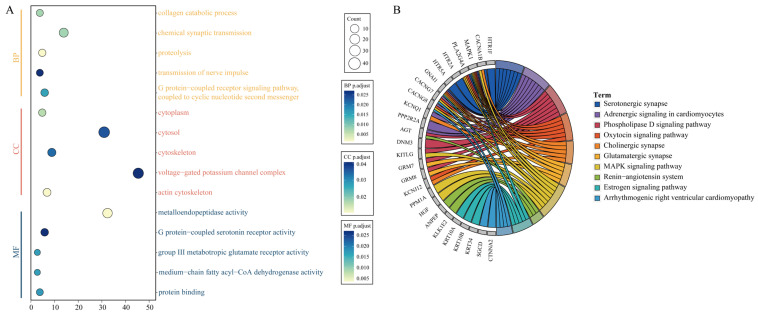
Functional enrichment of genes overlapping with CNVRs. (**A**) GO functional enrichment results. (**B**) KEGG functional enrichment results.

Using the DAVID online tool, GO and KEGG functional enrichment analyses were performed on the genes overlapping with CNVRs. The GO functional enrichment identified eleven biological processes, five cellular components, and eight molecular functions (FDR < 0.05) (Supplementary Table S7) (Figure 2A). The results were mainly associated with neural signaling, the cell structure and matrix, and metabolism and enzyme activity, for example, the transmission of nerve impulses (GO:0019226), actin cytoskeleton (GO:0015629), voltage-gated calcium channel activity (GO:0005245), and protein binding (GO:0005515). In addition, KEGG functional annotation revealed that 10 pathways were significantly enriched (FDR < 0.05) (Figure 2B). The KEGG pathways primarily involved neural synapse- and oxidative stress-related pathways (Supplementary Table S8), including Adrenergic signaling in cardiomyocytes (ecb04261), the MAPK signaling pathway (ecb04010), and the renin–angiotensin system (ecb04614), which were significantly enriched.

The CNVRs detected in this study were compared with the QTLs reported in the horse QTL database. The results showed that only five QTLs overlapped with CNVRs, including two Exterior Association QTLs, two Reproduction Association QTLs, and one Health Association QTL.

### 3.3. The Differential Expression Analysis of mRNA

**Figure 3:**
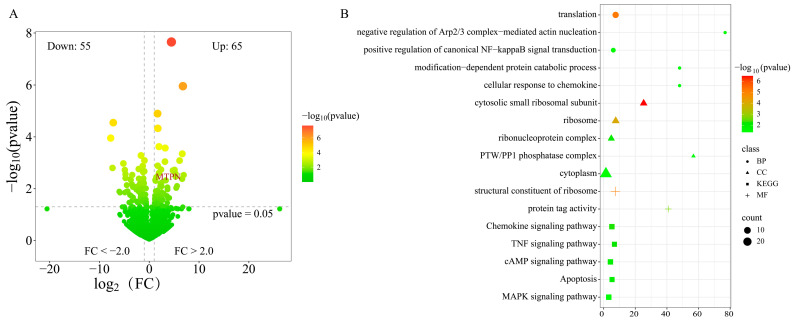
Differential genes and functional enrichment. (**A**) Volcano plot of differential mRNA. (**B**) Functional enrichment of differential genes.

To explore the regulatory role of mRNA in racing and non-racing populations, a total of 120 differentially expressed genes (DEGs) were identified, comprising 65 up-regulated genes and 55 down-regulated genes (Figure 3A) (Supplementary Table S9). A total of four genes overlapping with the CNVR region were identified, including three up-regulated genes, LOC100060228, LOC100056049, and MTPN (NC_009147.3: 90302401-90307100), and one down-regulated gene, LOC100053687. Among these, the frequency of MTPN in the competitive population was found to be seven times higher than in the non-competitive population. This gene was functionally enriched in the cytoplasm and protein binding (GO:0005737; GO:0005515). MTPN (Myotrophin) is closely associated with muscle development and cardiac function, and it is involved in regulating insulin secretion and glucose sensing. Functional enrichment analysis revealed that the overlapping genes between CNVR and mRNA were significantly enriched in the MAPK signaling pathway (ecb04010) in the KEGG database (Figure 3B). This pathway plays a crucial role in muscle growth, energy metabolism, and exercise adaptation.

### 3.4. CNVR-Based Population Differentiation

**Figure 4:**
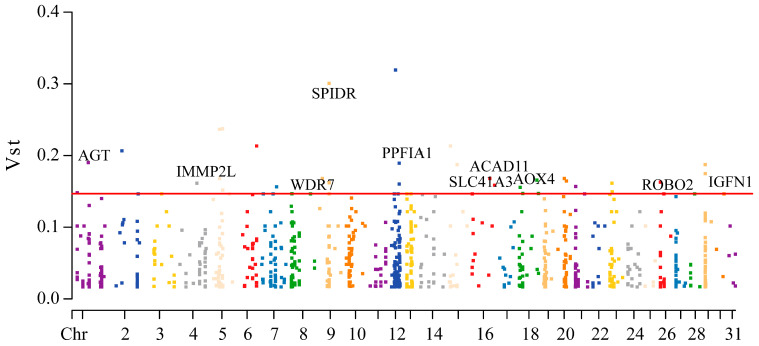
Genome-wide VST value plots for CNVRs. The red dashed line represents top 5% of VST value; *x*-axis representing the chromosomes and the *y*-axis representing the Vst values.

Vst analysis and *t*-tests were performed for each CNVR. To identify highly differentiated regions between the two groups, CNVRs with Vst values above the 95% threshold were selected as candidate regions. A total of 49 differential CNVRs were identified, and functional annotation was performed for the genes with Vst values above the threshold (Supplementary Table S6) (Figure 4). The results showed that a total of 47 genes exceeded the threshold. Functional analysis of the CNVR-associated genes above the threshold revealed six candidate genes—*AGT*, *IGFN1*, *IMMPL2*, *SLC41A3*, *AOX4*, and *ACAD11*. These genes are closely related to energy metabolism, muscle contraction, and muscle repair.

### 3.5. qPCR Validation of CNVRs

**Figure 5:**
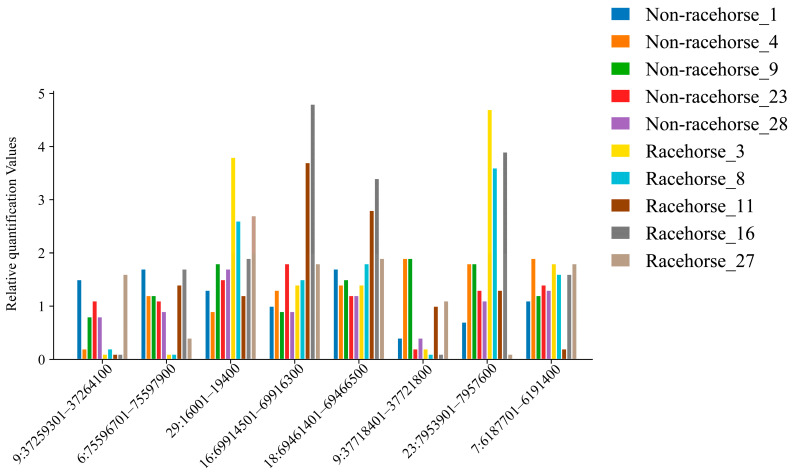
RT-PCR validation of selected CNVRs. The *y*-axis shows the relative quantification values obtained by RT-PCR, while the *x*-axis indicates the sample names in the different CNV regions.

Eight CNVRs were randomly selected, and the accuracy of these CNVRs was validated using RT-PCR in a randomly chosen set of 10 horse samples. Meanwhile, the mRNA levels of related genes were examined using qRT-PCR. The results indicated that the randomly selected CNVRs and mRNA sequencing results were consistent with the RT-PCR findings (Figure 5) (Supplementary Figure S2).

## 4. Discussion

Whole-genome copy number variation detection is an effective strategy for identifying potential key genes associated with traits by analyzing the genes overlapping with CNVRs. Compared to chip-based methods, whole-genome sequencing-based methods perform better. Therefore, by detecting CNVRs between the racehorse and non-racehorse groups of Grassland-Thoroughbred horses, we aim to identify candidate genes related to athletic performance, providing a theoretical basis for future breeding strategies of Grassland-Thoroughbred horses. Currently, CNVs have been identified in more than 45 different horse breeds, accounting for approximately 1–3% of their genomes. The number of CNVs in genes is higher than in intergenic regions. Compared to the reference genome, typical CNV sizes in horses range from 1 kb to 4.84 Mb. In this study, the frequency of “deletion events” was much higher than that of “duplication events”, a pattern consistent with observations in other species [32,33]. The distribution of CNVRs is similar to what has been observed in previously reported studies on horse breeds. The number of CNVRs shows a positive correlation with the length of autosomes, with the highest number of shared CNVRs found on Chr12 [34]. Among all CNVRs, those smaller than 100 Kb accounted for the highest proportion, while CNVRs larger than 400 Kb were the least frequent. This may be due to the fact that shorter CNVRs are more common in the horse genome.

Differences in CNVRs have successfully been used as a method to distinguish horse breeds [35]. In this study, we compared the CNVRs of the Grassland-Thoroughbred with those reported in previous studies, and our analysis showed that approximately 42.5% of the CNVRs overlapped with those from 16 other horse breeds [36], Further comparison with CNVRs reported in 11 previous studies revealed that approximately 62.3% of the CNVRs overlapped [32,34,35,37,38,39,40,41,42], while about 16.4% of the CNVRs were described for the first time (Supplementary Table S10), which may be attributed to the stricter quantitative control applied in this study. In terms of variability, this substantial difference among breeds supports the hypothesis that CNVRs can serve as reliable genetic markers for breed differentiation [35]. On the other hand, in most studies, the number of deletions usually exceeds the number of duplications, whereas our findings show the opposite. Previous CNVR studies have shown considerable variation, ranging from a single CNVR identified in just two individuals in Brandenburger’s study to 5350 CNVRs detected in 222 individuals in the Friesian study. In our study, a total of 982 CNVRs were identified, which is an intermediate number. However, such differences may be influenced by various factors, including detection methods, sample size, genotyping platforms, and the criteria used for CNVR identification in each study [43]. Therefore, further systematic studies are needed to accurately evaluate CNVR characteristics across different species.

Racehorses may have undergone intense artificial selection pressure, leading to the deletion or duplication of certain genes or regulatory regions. Analyzing the overlapping genes between mRNA and CNVR can reveal the impact of CNVs on gene expression and help identify key genes associated with specific traits. In the genomic region NC_009147.3: 90302401-90307100, a copy number duplication was observed, and the mRNA expression of *MTPN* was found to be upregulated in the competitive population. This suggests that the copy number variation in this region may contribute to the increased expression of *MTPN*, potentially influencing traits related to muscle development, cardiac function, and metabolic regulation in the competitive group. The gene *MTPN* is capable of regulating the growth of both cardiac and skeletal muscle cells. Shiraishi et al. observed that mice treated with *MTPN* exhibited increased body weight and more regular muscle fiber morphology compared to control mice, indicating that *MTPN* possesses muscle growth-promoting activity in mice. This finding underscores the potential role of *MTPN* in enhancing muscle development and its relevance to physiological adaptations in competitive populations [44]. Anderson et al. found that *MTPN* can induce cardiomyocyte hypertrophy and increase the expression level of dystrophin mRNA in the hearts of individuals with dilated cardiomyopathy [45]. Additionally, *MTPN* has a positive impact on skeletal muscle hypertrophy and myotube morphology [46]. In this study, *MTPN* exhibited upregulated mRNA expression within the duplicated CNVR region. This phenomenon suggests that *MTPN* upregulation may enhance muscle function by promoting muscle fiber hypertrophy and optimizing myotube morphology, thereby improving speed and endurance. Additionally, in the case of muscle injury in the competitive population, *MTPN* may play a crucial role in the repair process. However, its function requires further validation.

The distribution of CNVRs in mammalian genomes is not uniform, with some of them affecting key biological processes and playing an important role in adaptive evolution and phenotypic variation. GO and KEGG functional enrichment analyses revealed that some pathways are associated with energy metabolism and muscle function, which are related to athletic performance. The MAPK signaling pathway and the renin–angiotensin system are closely related to athletic traits. Endurance horses, during prolonged submaximal exercise, need to maintain a high metabolic level to ensure that muscles continuously receive sufficient energy support. The metabolic rate of endurance horses can increase 10 to 20 times, allowing them to efficiently utilize stored energy sources in the body. The renin–angiotensin system plays a crucial role in regulating blood pressure and fluid balance, which in turn affects the horse’s athletic performance during competitions [47]. The MAPK signaling pathway is activated during exercise and participates in carbohydrate and fat metabolism, cell proliferation, differentiation, and other processes through phosphorylation. Studies have shown that treadmill exercise in rodents [48], cycling exercise [49], and marathon running [50] all increase the phosphorylation of MAPK, with activation primarily occurring during high-intensity muscle contraction [51]. The functional genes associated with athletic performance screened from CNVRs may be related to the specific performance of Grassland-Thoroughbred horses in competitions. Although we integrated CNVRs into QTLs and identified five QTLs overlapping with CNVRs, unfortunately, no QTLs related to athletic performance were found.

In recent years, CNVs have gradually been recognized as a source of genomic variation and phenotypic variation. Similarly to SNPs, CNVs can be used to identify associations with genetic diseases and other complex traits. CNVs can be regulated through dosage effects, copy number effects, and positional effects. Numerous studies have shown that genes overlapping with CNVRs can influence traits in livestock. Selective sweep can reveal regions that have been influenced by environmental and artificial selection during local adaptation and domestication. By calculating pairwise Vst values, key CNVRs with significant differences between populations can be identified. *AGT* (angiotensinogen) can ensure the timely supply of oxygen and energy during exercise by enhancing the heart’s pumping ability and metabolic regulation. *AGT* interacts with renin to produce angiotensin I, which is further converted into angiotensin II (ANG II) by angiotensin-converting enzyme. ANG II is a potent vasoconstrictor, thereby playing a crucial role in blood pressure regulation [52,53]. The effect of *AGT* may stem from its regulation of ANG II levels. Its polymorphism, particularly a missense mutation, is widely used in sports research to study its impact on athletic performance [54,55]. The potential mechanisms of ANG II on muscle performance may include: a direct hypertrophic effect on skeletal muscles, as well as the redistribution of blood flow from type I fibers to the faster and more powerful type II fibers [54,56]. Multiple studies have shown that the *AGT* M235T polymorphism is more prevalent in strength athletes and is associated with better performance in explosive sports [57]. Muscle strength is often considered closely related to health. *IGFN1* (Immunoglobulin-Like Fibronectin Type III Domain-Containing 1) has been found to decrease with age, with higher expression levels in younger individuals. Additionally, *IGFN1* expression is upregulated in individuals who perform well in the “Timed Up and Go” test [58]. Other studies have shown that after an exercise training regimen, the mRNA levels of *IGFN1* in muscles increase [59]. *IGFN1* is essential for myoblast fusion and differentiation [60]. Functional association analysis has revealed that *IGFN1*, a kinase gene, is primarily involved in processes such as protein translation, skeletal muscle contraction, muscle development, skeletal muscle cytoskeleton, and intracellular calcium ion balance [61]. This suggests that *IGFN1* may play an important role in maintaining muscle function and athletic performance. The *IMMP2L* protein is a subunit of the mitochondrial inner membrane peptidase heterodimer complex, and defects in *IMMP2L* can lead to elevated mitochondrial ATP levels [62]. Multiple regression analysis suggests that *IMMP2L* may have a negative impact on the abundance of *SLC25A25* protein. In acute endurance exercise experiments, *IMMP2L* expression significantly decreases post exercise, while *SLC25A25* transcript levels increase immediately after exercise. The deletion of *IMMP2L* can lead to a reduction in mitochondrial ATP levels, which may play a role in mediating acute endurance exercise [63,64]. *IMMP2L* is closely associated with exercise-induced mitochondrial adaptation by regulating mitochondrial ATP levels and the expression of *SLC25A25*. Its post-exercise downregulation may help promote the efficiency of mitochondrial energy metabolism, thereby meeting the energy demands of exercise. In mitochondria, Mg^2+^ not only chelates and stabilizes ATP but also serves as a cofactor for enzymes involved in cellular respiration and energy production [65]. *SLC41A3* is primarily expressed on the mitochondrial membrane and mediates the efflux of mitochondrial Mg^2+^ [66]. The role of Mg^2+^ in energy metabolism partially interferes with the stimulatory effect of Ca^2+^ on energy metabolism and its impact on mitochondrial Ca^2+^ transport, which is particularly important in excitable cells such as neurons and muscle cells [67]. Neurobehavioral abnormalities, particularly motor coordination defects associated with ataxia, were detected in conditional knockout mice of *SLC41A3* (http://www.mousephenotype.org/, accessed on 25 November 2024) [68]. In diabetic patients, three months of cycling exercise led to a decrease in magnesium levels, and the expression of *SLC41A3* was downregulated post exercise [69]. Muscle fiber strength in young *AOX4* knockout mice exhibited mild defects, which became more pronounced with age in mature mice, and the tibialis muscle fiber diameter was smaller compared to the control group. Knockout of *AOX4* leads to changes in energy homeostasis, with a significant reduction in physical activity during the circadian cycle, which becomes more pronounced over time [70]. Cells utilize fatty acids as an energy source through the β-oxidation pathway, which occurs in the mitochondria. Acyl-CoA dehydrogenase catalyzes the hydrolysis of thioester bonds in various CoA esters. ACADs are a class of mitochondrial flavin enzymes involved in the catabolism of fatty acids (β-oxidation) and amino acids. In mice, the mRNA expression of *ACAD11*, which is associated with fat oxidation, significantly increases during exercise [71].

Due to the adaptive evolution undergone by the Grassland-Thoroughbred during long-term artificial selection, CNV detection in the competitive population identified six candidate genes: *AGT*, *IGFN1*, *IMMPL2*, *SLC41A3*, *AOX4*, and *ACAD11*. These genes may have undergone strong selective pressure related to the population’s athletic traits, contributing to better performance in competitions, However, further functional validation is still needed. In the future, comparing with renowned racehorse breeds such as Thoroughbreds and Arabians will help better identify potential genetic markers, advancing the application of the Grassland-Thoroughbred in future breeding and enhancing its athletic performance and overall quality.

## 5. Conclusions

This study investigated the genomic features and structural variations between racehorses and non-racehorses of the Grassland-Thoroughbred horse breed using whole-genome resequencing and transcriptomic data, identifying 982 CNVRs. We also identified copy number variation genes potentially related to the athletic traits of the Grassland-Thoroughbred, including *AGT*, *IGFN1*, *IMMPL2*, *SLC41A3*, *AOX4*, and *ACAD11*, though further validation is required. Transcriptomic analysis revealed 120 differentially expressed genes, with *MTPN* expressed in both CNVR-overlapping genes and mRNA. Both CNVR-overlapping genes and differentially expressed genes were enriched in the MAPK signaling pathway, indicating that genetic variation driven by artificial selection has a significant effect at the CNV level. This study provides valuable resources for structural variation research in horse genomes and offers new insights into the genetic basis of athletic adaptability and performance, laying a theoretical foundation for breeding strategies aimed at improving the performance and adaptability of local competitive horse breeds.

## Data Availability

Sequence data that support the findings of this study have been deposited in the National Center for Biotechnology Information with the primary accession codes PRJNA11630453 and PRJNA1189315.

## Supplementary Materials

The following supporting information can be downloaded at: https://www.mdpi.com/article/10.3390/ani15101458/s1, Supplementary Table S1: Sampling and competition records of grassland-thoroughbreds; Supplementary Table S2: Primers of qPCR; Supplementary Table S3: The statistical table of sequencing results for each sample; Supplementary Table S4: Detailed CNV information for each sample; Supplementary Table S5: Detailed information on the detected CNVRs and the differential CNVRs between the racing and non-racing groups; Supplementary Table S6: Vst, ANOVA, and annotated gene information; Supplementary Table S7: GO functional enrichment of genes overlapping with CNVRs; Supplementary Table S8: KEGG functional enrichment of genes overlapping with CNVRs; Supplementary Table S9: Differentially expressed genes and functional enrichment; Supplementary Table S10 CNVR comparative information; Supplementary Figure S1: Breeding route and phylogenetic relationship heatmap of the Grassland-Thoroughbreds; Supplementary Figure S2: qPCR validation of selected differentially expressed genes.

## References

[1] Chien P.M. Tourism and Hospitality in the Contemporary World: Trends, Changes & Complexity Proceedings of the 24th Annual Conference CAUTHE 2014. Proceedings of the CAUTHE Conference 2014.

[2] Worthington A.C. (2007). National exuberance: A note on the Melbourne Cup effect in Australian stock returns. Econ. Pap. A J. Appl. Econ. Policy.

[3] Narayan P.K., Smyth R. (2004). The race that stops a nation: The demand for the Melbourne Cup. Econ. Rec..

[4] Mostafavi A., Fozi M.A., Koshkooieh A.E., Mohammadabadi M., Babenko O.I., Klopenko N.I. (2019). Effect of LCORL gene polymorphism on body size traits in horse populations. Acta Scientiarum. Anim. Sci..

[5] Davis M. (2010). When Things Get Dark: A Mongolian Winter’s Tale.

[6] Qi B. (2002). Xilin Gol League Animal Husbandry Chronicle.

[7] Zhang F., Gu W., Hurles M.E., Lupski J.R. (2009). Copy number variation in human health, disease, and evolution. Annu. Rev. Genom. Hum. Genet..

[8] Stranger B.E., Forrest M.S., Dunning M., Ingle C.E., Beazley C., Thorne N., Redon R., Bird C.P., De Grassi A., Lee C. (2007). Relative impact of nucleotide and copy number variation on gene expression phenotypes. Science.

[9] Conrad D.F., Pinto D., Redon R., Feuk L., Gokcumen O., Zhang Y., Aerts J., Andrews T.D., Barnes C., Campbell P. (2010). Origins and functional impact of copy number variation in the human genome. Nature.

[10] Scherer S.W., Lee C., Birney E., Altshuler D.M., Eichler E.E., Carter N.P., Hurles M.E., Feuk L. (2007). Challenges and standards in integrating surveys of structural variation. Nat. Genet..

[11] Freeman J.L., Perry G.H., Feuk L., Redon R., McCarroll S.A., Altshuler D.M., Aburatani H., Jones K.W., Tyler-Smith C., Hurles M.E. (2006). Copy number variation: New insights in genome diversity. Genome Res..

[12] Sebat J., Lakshmi B., Troge J., Alexander J., Young J., Lundin P., Manér S., Massa H., Walker M., Chi M. (2004). Large-scale copy number polymorphism in the human genome. Science.

[13] Clop A., Vidal O., Amills M. (2012). Copy number variation in the genomes of domestic animals. Anim. Genet..

[14] Conrad D.F., Bird C., Blackburne B., Lindsay S., Mamanova L., Lee C., Turner D.J., Hurles M.E. (2010). Mutation spectrum revealed by breakpoint sequencing of human germline CNVs. Nat. Genet..

[15] Stankiewicz P., Lupski J.R. (2010). Structural variation in the human genome and its role in disease. Annu. Rev. Med..

[16] Mazuet C., Legeay C., Sautereau J., Bouchier C., Criscuolo A., Bouvet P., Trehard H., Jourdan Da Silva N., Popoff M. (2017). Characterization of Clostridium Baratii Type F Strains Responsible for an Outbreak of Botulism Linked to Beef Meat Consumption in France. PLoS Curr..

[17] Chen S., Zhou Y., Chen Y., Gu J. (2018). fastp: An ultra-fast all-in-one FASTQ preprocessor. Bioinformatics.

[18] Wingett S.W., Andrews S. (2018). FastQ Screen: A tool for multi-genome mapping and quality control. F1000Res.

[19] Li H., Durbin R. (2009). Fast and accurate short read alignment with Burrows-Wheeler transform. Bioinformatics.

[20] Li H., Handsaker B., Wysoker A., Fennell T., Ruan J., Homer N., Marth G., Abecasis G., Durbin R., Subgroup G.P.D.P. (2009). The sequence alignment/map format and SAMtools. Bioinformatics.

[21] Bathke J., Lühken G. (2021). OVarFlow: A resource optimized GATK 4 based Open source Variant calling workFlow. BMC Bioinform..

[22] Purcell S., Neale B., Todd-Brown K., Thomas L., Ferreira M.A., Bender D., Maller J., Sklar P., De Bakker P.I., Daly M.J. (2007). PLINK: A tool set for whole-genome association and population-based linkage analyses. Am. J. Hum. Genet..

[23] Abyzov A., Urban A.E., Snyder M., Gerstein M. (2011). CNVnator: An approach to discover, genotype, and characterize typical and atypical CNVs from family and population genome sequencing. Genome Res..

[24] Pierce M.D., Dzama K., Muchadeyi F.C. (2018). Genetic diversity of seven cattle breeds inferred using copy number variations. Front. Genet..

[25] Kim D., Langmead B., Salzberg S.L. (2015). HISAT: A fast spliced aligner with low memory requirements. Nat. Methods.

[26] Kovaka S., Zimin A.V., Pertea G.M., Razaghi R., Salzberg S.L., Pertea M. (2019). Transcriptome assembly from long-read RNA-seq alignments with StringTie2. Genome Biol..

[27] Pertea M., Kim D., Pertea G.M., Leek J.T., Salzberg S.L. (2016). Transcript-level expression analysis of RNA-seq experiments with HISAT, StringTie and Ballgown. Nat. Protoc..

[28] Love M.I., Huber W., Anders S. (2014). Moderated estimation of fold change and dispersion for RNA-seq data with DESeq2. Genome Biol..

[29] Huang D.W., Sherman B.T., Lempicki R.A. (2009). Systematic and integrative analysis of large gene lists using DAVID bioinformatics resources. Nat. Protoc..

[30] Quinlan A.R., Hall I.M. (2010). BEDTools: A flexible suite of utilities for comparing genomic features. Bioinformatics.

[31] Aleman M., Nieto J.E. (2010). Gene expression of proteolytic systems and growth regulators of skeletal muscle in horses with myopathy associated with pituitary pars intermedia dysfunction. Am. J. Vet. Res..

[32] Doan R., Cohen N., Harrington J., Veazy K., Juras R., Cothran G., McCue M.E., Skow L., Dindot S.V. (2012). Identification of copy number variants in horses. Genome Res..

[33] Ghosh S., Das P., McQueen C., Gerber V., Swiderski C., Lavoie J.P., Chowdhary B., Raudsepp T. (2016). Analysis of genomic copy number variation in equine recurrent airway obstruction (heaves). Anim. Genet..

[34] Wang W., Wang S., Hou C., Xing Y., Cao J., Wu K., Liu C., Zhang D., Zhang L., Zhang Y. (2014). Genome-wide detection of copy number variations among diverse horse breeds by array CGH. PLoS ONE.

[35] Solé M., Ablondi M., Binzer-Panchal A., Velie B.D., Hollfelder N., Buys N., Ducro B.J., François L., Janssens S., Schurink A. (2019). Inter-and intra-breed genome-wide copy number diversity in a large cohort of European equine breeds. BMC Genom..

[36] Tang X., Zhu B., Ren R., Chen B., Li S., Gu J. (2023). Genome-wide copy number variation detection in a large cohort of diverse horse breeds by whole-genome sequencing. Front. Vet. Sci..

[37] Schurink A., da Silva V.H., Velie B.D., Dibbits B.W., Crooijmans R.P., Franҫois L., Janssens S., Stinckens A., Blott S., Buys N. (2018). Copy number variations in Friesian horses and genetic risk factors for insect bite hypersensitivity. BMC Genet..

[38] Dupuis M.-C., Zhang Z., Durkin K., Charlier C., Lekeux P., Georges M. (2013). Detection of copy number variants in the horse genome and examination of their association with recurrent laryngeal neuropathy. Anim. Genet..

[39] Pawlina-Tyszko K., Gurgul A., Szmatoła T., Ropka-Molik K., Semik-Gurgul E., Klukowska-Rötzler J., Koch C., Mählmann K., Bugno-Poniewierska M. (2017). Genomic landscape of copy number variation and copy neutral loss of heterozygosity events in equine sarcoids reveals increased instability of the sarcoid genome. Biochimie.

[40] Kader A., Liu X., Dong K., Song S., Pan J., Yang M., Chen X., He X., Jiang L., Ma Y. (2016). Identification of copy number variations in three Chinese horse breeds using 70K single nucleotide polymorphism BeadChip array. Anim. Genet..

[41] Ghosh S., Qu Z., Das P.J., Fang E., Juras R., Cothran E.G., McDonell S., Kenney D.G., Lear T.L., Adelson D.L. (2014). Copy number variation in the horse genome. PLoS Genet..

[42] Wang M., Liu Y., Bi X., Ma H., Zeng G., Guo J., Guo M., Ling Y., Zhao C. (2022). Genome-wide detection of copy number variants in Chinese indigenous horse breeds and verification of CNV-overlapped genes related to heat adaptation of the Jinjiang horse. Genes..

[43] Metzger J., Philipp U., Lopes M.S., da Camara Machado A., Felicetti M., Silvestrelli M., Distl O. (2013). Analysis of copy number variants by three detection algorithms and their association with body size in horses. BMC Genom..

[44] Shiraishi S., Nakamura Y.-N., Iwamoto H., Haruno A., Sato Y., Mori S., Ikeuchi Y., Chikushi J., Hayashi T., Sato M. (2006). S-myotrophin promotes the hypertrophy of skeletal muscle of mice in vivo. Int. J. Biochem. Cell Biol..

[45] Anderson K.M., Berrebi-Bertrand I., Kirkpatrick R.B., McQueney M.S., Underwood D.C., Rouanet S., Chabot-Fletcher M. (1999). cDNA sequence and characterization of the gene that encodes human myotrophin/V-1 protein, a mediator of cardiac hypertrophy. J. Mol. Cell. Cardiol..

[46] Bordbar F., Jensen J., Du M., Abied A., Guo W., Xu L., Gao H., Zhang L., Li J. (2020). Identification and validation of a novel candidate gene regulating net meat weight in Simmental beef cattle based on imputed next-generation sequencing. Cell Prolif..

[47] De Mello Costa M., Anderson G., Davies H., El-Hage C., Slocombe R. (2010). Circulating angiotensin converting enzyme in endurance horses: Effect of exercise on blood levels and its value in predicting performance. Equine Vet. J..

[48] Nader G.A., Esser K.A. (2001). Intracellular signaling specificity in skeletal muscle in response to different modes of exercise. J. Appl. Physiol..

[49] Widegren U., Jiang X.J., Krook A., Chibalin A.V., Björnholm M., Tally M., Roth R.A., Henriksson J., Wallberg-Henriksson H., Zierath J.R. (1998). Divergent effects of exercise on metabolic and mitogenic signaling pathways in human skeletal muscle. FASEB J..

[50] Yu M., Blomstrand E., Chibalin A.V., Krook A., Zierath J.R. (2001). Marathon running increases ERK1/2 and p38 MAP kinase signalling to downstream targets in human skeletal muscle. J. Physiol..

[51] Kramer H.F., Goodyear L.J. (2007). Exercise, MAPK, and NF-κB signaling in skeletal muscle. J. Appl. Physiol..

[52] Rauramaa R., Kuhanen R., Lakka T.A., Väisänen S.B., Halonen P., Alén M., Rankinen T., Bouchard C. (2002). Physical exercise and blood pressure with reference to the angiotensinogen M235T polymorphism. Physiol. Genom..

[53] Corvol P., Jeunemaitre X. (1997). Molecular genetics of human hypertension: Role of angiotensinogen. Endocr. Rev..

[54] Jones A., Woods D.R. (2003). Skeletal muscle RAS and exercise performance. Int. J. Biochem. Cell Biol..

[55] Ben-Zaken S., Eliakim A., Nemet D., Meckel Y. (2019). Genetic Variability Among Power Athletes: The Stronger vs. the Faster. J. Strength. Cond. Res..

[56] Aleksandra Z., Zbigniew J., Waldemar M., Agata L.-D., Mariusz K., Marek S., Agnieszka M.-S., Piotr Ż., Krzysztof F., Grzegorz T. (2016). The AGT gene M235T polymorphism and response of power-related variables to aerobic training. J. Sports Sci. Med..

[57] Zarebska A., Sawczyn S., Kaczmarczyk M., Ficek K., Maciejewska-Karlowska A., Sawczuk M., LeoNska-Duniec A., Eider J., Grenda A., Cieszczyk P. (2013). Association of rs699 (M235T) polymorphism in the AGT gene with power but not endurance athlete status. J. Strength. Cond. Res..

[58] Perez K., Ciotlos S., McGirr J., Limbad C., Doi R., Nederveen J.P., Nilsson M.I., Winer D.A., Evans W., Tarnopolsky M. (2022). Single nuclei profiling identifies cell specific markers of skeletal muscle aging, frailty, and senescence. Aging.

[59] Riedl I., Yoshioka M., Nishida Y., Tobina T., Paradis R., Shono N., Tanaka H., St-Amand J. (2010). Regulation of skeletal muscle transcriptome in elderly men after 6 weeks of endurance training at lactate threshold intensity. Exp. Gerontol..

[60] Li X., Baker J., Cracknell T., Haynes A.R., Blanco G. (2017). IGFN1_v1 is required for myoblast fusion and differentiation. PLoS ONE.

[61] Kilpinen S., Ojala K., Kallioniemi O. (2010). Analysis of kinase gene expression patterns across 5681 human tissue samples reveals functional genomic taxonomy of the kinome. PLoS ONE.

[62] Lu B., Poirier C., Gaspar T., Gratzke C., Harrison W., Busija D., Matzuk M.M., Andersson K.-E., Overbeek P.A., Bishop C.E. (2008). A mutation in the inner mitochondrial membrane peptidase 2-like gene (Immp2l) affects mitochondrial function and impairs fertility in mice. Biol. Reprod..

[63] Sako H., Yada K., Suzuki K. (2016). Genome-Wide Analysis of Acute Endurance Exercise-Induced Translational Regulation in Mouse Skeletal Muscle. PLoS ONE.

[64] Anunciado-Koza R.P., Zhang J., Ukropec J., Bajpeyi S., Koza R.A., Rogers R.C., Cefalu W.T., Mynatt R.L., Kozak L.P. (2011). Inactivation of the mitochondrial carrier SLC25A25 (ATP-Mg^2+^/Pi transporter) reduces physical endurance and metabolic efficiency in mice. J. Biol. Chem..

[65] Volpe S.L. (2013). Magnesium in disease prevention and overall health. Adv. Nutr..

[66] Mastrototaro L., Smorodchenko A., Aschenbach J.R., Kolisek M., Sponder G. (2016). Solute carrier 41A3 encodes for a mitochondrial Mg(2+) efflux system. Sci. Rep..

[67] Williams G.S., Boyman L., Lederer W.J. (2015). Mitochondrial calcium and the regulation of metabolism in the heart. J. Mol. Cell. Cardiol..

[68] Fleig A., Schweigel-Röntgen M., Kolisek M. (2013). Solute carrier family SLC41: What do we really know about it?. Wiley Interdiscip. Rev. Membr. Transp. Signal..

[69] Chiang Y.-F., Chen H.-Y., Lee I.-T., Chien L.-S., Huang J.-H., Kolisek M., Cheng F.-C., Tsai S.-W. (2019). Magnesium-responsive genes are downregulated in diabetic patients after a three-month exercise program on a bicycle ergometer. J. Chin. Med. Assoc..

[70] Terao M., Barzago M.M., Kurosaki M., Fratelli M., Bolis M., Borsotti A., Bigini P., Micotti E., Carli M., Invernizzi R.W. (2016). Mouse aldehyde-oxidase-4 controls diurnal rhythms, fat deposition and locomotor activity. Sci. Rep..

[71] Slocum N., Durrant J.R., Bailey D., Yoon L., Jordan H., Barton J., Brown R.H., Clifton L., Milliken T., Harrington W. (2013). Responses of brown adipose tissue to diet-induced obesity, exercise, dietary restriction and ephedrine treatment. Exp. Toxicol. Pathol..

