# Supplementary figures and images for: Whole-Genome Resequencing Analysis of Copy Number Variations Associated with Athletic Performance in Grassland-Thoroughbred
Source: Animals (Basel). 2025 May 18;15(10):1458. doi: 10.3390/ani15101458 (PMC12108297; doi:10.3390/ani15101458)

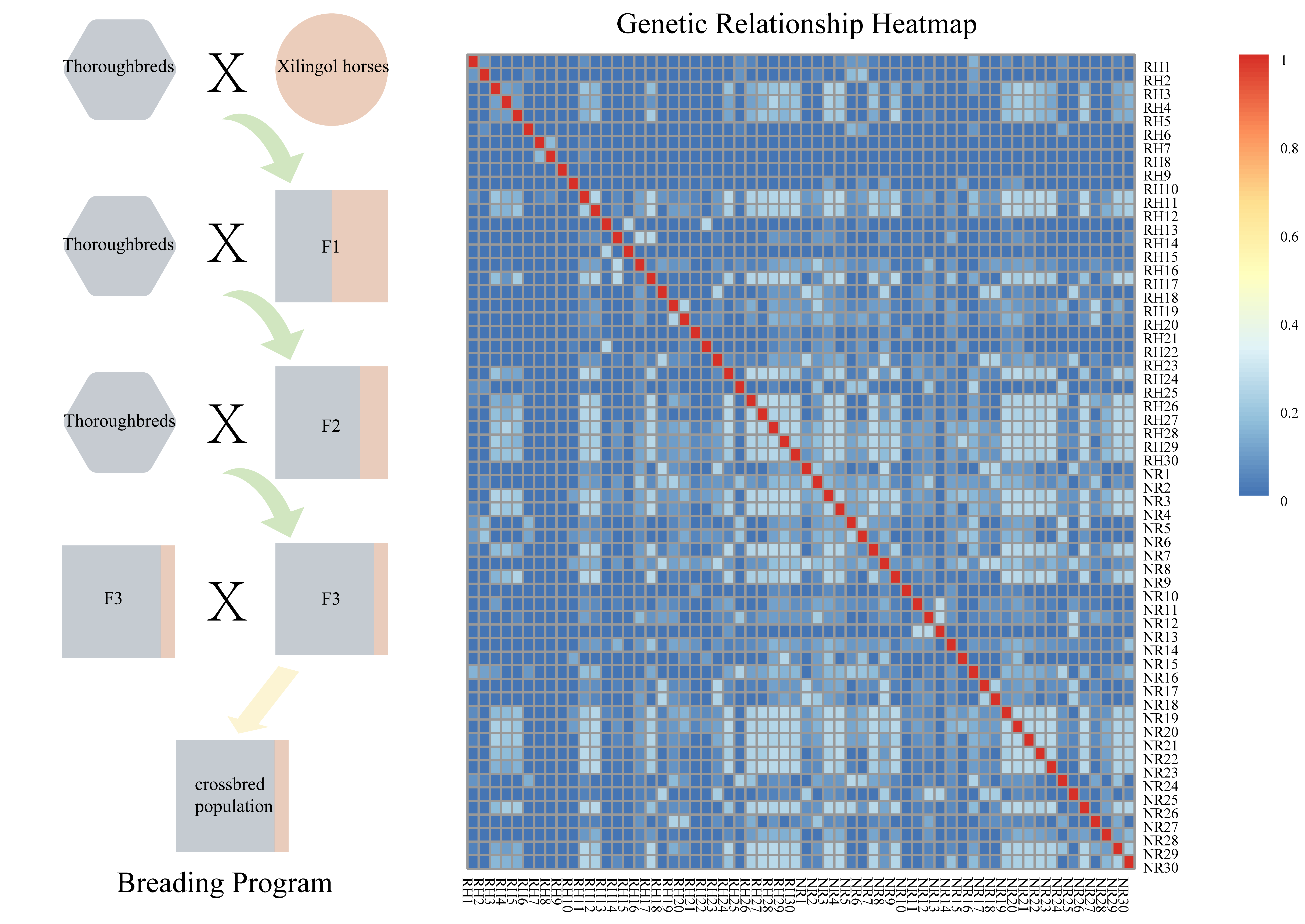

Supplement: Supplementary file 1 [file animals-15-01458-s001.zip › Supplementary Figure S1 Breeding route of the Grassland Thoroughbreds.png]
